# An Audit of Compliance with Trust Guidelines for Post-Incident Medical Reviews of Patients who have Ligatured on Acute Inpatient Psychiatric Wards in CWP NHS Foundation Trust, using a Sample of Data from the period between January 2022 and May 2024

**DOI:** 10.1192/j.eurpsy.2025.2161

**Published:** 2025-08-26

**Authors:** A. Brown, K. Clark, Z. Wrightson, M. Godsiff, S. Button

**Affiliations:** 1 Psychiatry, Cheshire and Wirral Partnership NHS Foundation Trust, Cheshire and Wirral, United Kingdom

## Abstract

**Introduction:**

Immediate physical assessment and management of patients on psychiatric wards who have been ligaturing is not standardised across the UK. There is little published research or literature on what is needed in terms of medical input and these incidents are usually initially assessed/managed by non-medical staff who are on site at the time of any such incidents. Within CWP NHS Foundation Trust, we have local guidelines (SOP 13 ‘The Management of ligatures in Mental Health and Learning Disability Services’) advising that any inpatient on the adult/older adult psychiatry wards who has ligatured should be seen by a doctor for a medical review,

**Objectives:**

To review a sample of recorded inpatient ligature incidents to see if Trust guidelines were being adhered to. We hope to use the findings from this audit to review the current guidelines and assess whether or not the additional medical reviews add to or change clinical management already instigated by ward staff. This may be more of an issue when medical staff cover is limited e.g. out of hours.

**Methods:**

We accessed recorded ligaturing incidents on adult and older adult inpatient psychiatry wards across our Trust (accessing the ‘Datix’ reporting system) from the period starting 1st January 2022 to 31st May 2024. In total, there were 1127 and we took a sample of 112 picked using a random number generator. We reviewed the documentation from the incident to confirm how many had had a medical review after the incident, how long after the incident they were seen and whether or not the medical review had changed management following the incident.

**Results:**

Approximately 50% of patients had had a medical review post ligature incident. Approximately 4% of patient ligaturing (5/112) or 9% of those who received a medical review (5/55) had new management instigated as a result of the medic review. On review of these cases, there was limited medical input needed including application of steristrips for wound care and asking for ambulance transfer to acute hospital for CT head following seizure after ligaturing. There were no serious harm outcomes from the patients we reviewed in our sample.

**Conclusions:**

Whether or not the we can review guidlines can be reviewed in light of the data is to be discussed following presentation of our results to the Trust. It appears that the initial managment plan, instigated by ward staff, has usually been appropriate and when additional input has been given by the medic on site at review, this has not been felt to have been critical in optimising patient safety. It would still be possible for a medic to review patients when felt by ward staff to be necessary even if guidelines were changed to suggest it was not mandatory for patients to be seen by a medic.

**Disclosure of Interest:**

None Declared

